# Antioxidant and Signal-Modulating Effects of Brown Seaweed-Derived Compounds against Oxidative Stress-Associated Pathology

**DOI:** 10.1155/2021/9974890

**Published:** 2021-07-10

**Authors:** Rahima Begum, Saurav Howlader, A. N. M. Mamun-Or-Rashid, S. M. Rafiquzzaman, Ghulam Md Ashraf, Ghadeer M. Albadrani, Amany A. Sayed, Ilaria Peluso, Mohamed M. Abdel-Daim, Md. Sahab Uddin

**Affiliations:** ^1^Department of Environmental Medical Biology, Wonju College of Medicine, Yonsei University, Wonju, Gangwon-do, 26426, Republic of Korea; ^2^Department of Pharmacology and Pharmaco Genomics Research Centre (PGRC), Inje University College of Medicine, Busan, Republic of Korea; ^3^Anti-Aging Medical Research Center and Glycative Stress Research Center, Graduate School of Life and Medical Sciences, Doshisha University, 1-3 Tatara Miyakodani, Kyotanabe, Kyoto 610-0394, Japan; ^4^Department of Fisheries Biology & Aquatic Environment, Bangabandhu Sheikh Mujibur Rahman Agricultural University (BSMRAU), Gazipur 1706, Bangladesh; ^5^Pre-Clinical Research Unit, King Fahd Medical Research Center, King Abdulaziz University, Jeddah, Saudi Arabia; ^6^Department of Medical Laboratory Technology, Faculty of Applied Medical Sciences, King Abdulaziz University, Jeddah, Saudi Arabia; ^7^Department of Biology, College of Science, Princess Nourah bint Abdulrahman University, Riyadh 11474, Saudi Arabia; ^8^Zoology Department, Faculty of Science, Cairo University, Giza 12613, Egypt; ^9^Research Centre for Food and Nutrition, Council for Agricultural Research and Economics (CREA-AN), 00142 Rome, Italy; ^10^Pharmacology Department, Faculty of Veterinary Medicine, Suez Canal University, Ismailia 41522, Egypt; ^11^Department of Pharmacy, Southeast University, Dhaka, Bangladesh; ^12^Pharmakon Neuroscience Research Network, Dhaka, Bangladesh

## Abstract

The biological and therapeutic properties of seaweeds have already been well known. Several studies showed that among the various natural marine sources of antioxidants, seaweeds have become a potential source of antioxidants because of their bioactive compounds. Most of the metabolic diseases are caused by oxidative stress. It is very well known that antioxidants have a pivotal role in the treatment of those diseases. Recent researches have revealed the potential activity of seaweeds as complementary medicine, which have therapeutic properties for health and disease management. Among the seaweeds, brown seaweeds (Phaeophyta) and their derived bioactive substances showed excellent antioxidant properties than other seaweeds. This review focuses on brown seaweeds and their derived major bioactive compounds such as sulfated polysaccharide, polyphenol, carotenoid, and sterol antioxidant effects and molecular mechanisms in the case of the oxidative stress-originated disease. Antioxidants have a potential role in the modification of stress-induced signaling pathways along with the activation of the oxidative defensive pathways. This review would help to provide the basis for further studies to researchers on the potential antioxidant role in the field of medical health care and future drug development.

## 1. Introduction

Brown seaweeds are photosynthetic aquatic algae which belong to the domain of Eukarya, kingdom of Chromista, and class of Phaeophyta [1]. There are 1500 species of brown seaweeds all over the world [2]. In particular, in Asian countries, seaweed has been used as a traditional herbal medicine for the treatment of gastrointestinal problems, cough, boils, ulcers, asthma, cough, and headache as well as vegetables too [3]. Lately, several studies revealed that dietary seaweeds not only are a good source of carbohydrates, dietary fiber, proteins and peptides, vitamins, minerals, and fats but also contain a large concentration of functionally bioactive compounds such as carotenoids, polysaccharides, polyphenols, and sterols, which have potential antioxidant properties as well as antimicrobial, anticoagulant, antithrombotic, anti-inflammatory, antitumor, and antiviral properties for several diseases [4–6]. Therefore, nowadays, seaweeds have been paid attention to for the development of medicine, food, cosmetics, dietary supplements, fertilizer, and bioenergy [7].

Oxidative stress is widely involved in the development of many chronic diseases such as cardiovascular disease, neurodegenerative, cardiovascular, cancer, inflammation, diabetes, obesity, and aging and many of the elderly diseases [8–10]. Antioxidants are the only therapeutic molecules capable of blocking oxidative stress by their excellent reactive oxygen species (ROS) scavenging activity with low or no toxicity [11]. An antioxidant optimizes the human physiological function that helps to protect against disease or disease progression as well as maintains a healthy state [12]. Naturally, endogenous antioxidants are present in our body; the additional exogenous supplement can also be obtained from various natural sources and chemically synthetic antioxidants such as butylated hydroxyanisole (BHA), butylated hydroxytoluene (BHT), and tert-butylhydroquinone (TBHQ) [13–15]. However, studies proved chemically synthesized antioxidants to be toxic and carcinogenic, whether the natural antioxidant is safe, more effective, and easily absorbed by the body [15]. The dietary antioxidants such as *α*-tocopherol, ascorbic acid, carotenoids, amino acids, peptides, proteins, flavonoids, and other phenolic compounds were found effective in the boosting of the antioxidant mechanism [16]. The marine world is a rich source of bioactive molecules. Among the various natural sources of antioxidants, marine seaweeds and their bioactive compounds are gaining worldwide attention [4, 17] in industry and drugs since 1980 [18]. Numerous evidences have shown that the brown seaweed-derived compounds are capable of improving the oxidative stress-induced diseases including neurodegenerative disease [19], cardiovascular-associated disorders [20], and obesity [21], as well as cancer protection [22]. But unfortunately, we do not have much information about the antioxidant effects and molecular mechanisms of brown seaweeds in oxidative stress diseases.

It has been reported that brown seaweeds have higher antioxidant properties comparatively than red and green seaweeds [23]. Brown seaweeds contain one of the most abundant pigment carotenoid compounds, fucoxanthin, and are estimated to contain around 10% of total carotenoids found in nature [24, 25]. Fucoxanthin has a great antioxidant activity as well as anti-inflammatory, antidiabetic, antiphotoaging, and neuroprotective properties [26]. Polyphenol compounds in brown algae such as phlorotannins are the unique and most dominant complex group of polymers named phloroglucinol (1,3, 5-trihydroxybenzene). It is mainly formed as secondary metabolites in the acetate-malonate pathway. Phlorotannin bioactive compounds exist as soluble compounds or cell-bound forms, mainly produced by brown seaweeds to protect themselves from herbivores and stress conditions, minimizing the oxidative damage caused by nutrient deprivation and ultraviolet radiation. Phlorotannins isolated from brown algae *Ecklonia cava*, one of the most abundant sources of polyphenolic compounds, have been revealed to have higher antioxidant activity *in vitro* and *in viv*o [6, 7, 20, 27]. Another bioactive compound of brown seaweeds is sulfated polysaccharides (SPs), which generally comprise a high content of major groups of sugar, e.g., fucose, galactose, uronic acid, and sulfate [28]. The antioxidant activities of SPs depend on their major sugar, degree of sulfating, low molecular weight, and glycosidic branching [29–31]. The major SPs found in brown seaweeds are fucoidan [32, 33]. Over the past decade, the antioxidant compound sterols called fucosterol have shown great antioxidant property as well as important contributions to human health and wellness [34].

This present review summarizes and discusses brown seaweeds and their major bioderived compound's role as an antioxidant in oxidative stress and their action in the management of disease-related signaling pathways. In Table 1, we show the antioxidant activity of brown seaweed-derived major compounds from the literature review and research articles.

## 2. Oxidative Stress, Antioxidant Defense, and Signal Transduction Pathway in the Cell

**Figure 1:**
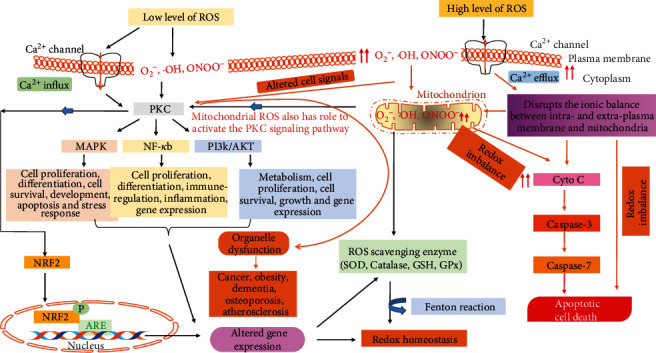
Redox homeostasis with cell signaling pathway under lower and higher levels of ROS.

**Figure 2:**
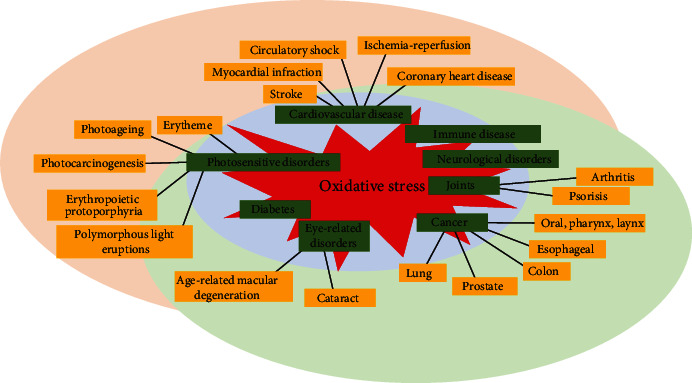
Examples of ROS and oxidative stress-induced diseases.

Generally, oxidative stress is the imbalance between the production of ROS and the body's own antioxidant defensive system [50]. ROS, usually generated through various extracellular and intracellular processes, are involved in cellular growth, differentiation, progression, and cell death as well as cell signaling [51, 52]. Excessive production of ROS causes oxidative stress and modifies the structure of the cellular macromolecules such as lipids, proteins, nucleic acids, and DNA. Consequently, the cellular and biological functions are inactivated with the modification of cellular signaling. Generally, under the physiological condition, ROS maintain the body's homeostasis by the regulation of several cell signaling pathways which are involved in cellular processes such as mitogen-activated protein kinase (MAPK), nuclear factor-kappa B (NF-*κ*B), and phosphatidylinositol 3‑kinase (PI3K) (Figure 1) as well as the defensive pathway nuclear factor erythroid 2–related factor 2 (Nrf2) signaling; therefore, ROS is considered as a secondary messenger for the activation of cellular signaling [50, 53–55]. Nrf2 is a redox-sensitive transcription factor that binds to antioxidant response elements (ARE) to regulate the expression of antioxidant enzymes that protect the cell against oxidative damage by inducing the antioxidant enzymes. Moreover, excessive ROS modulates the cellular antioxidant defense system that has eventually altered the normal physiological signal and switched to the apoptosis or cell death signals. Therefore, oxidative stress is to be involved in the development of many diseases including cancer, Parkinson's disease, Alzheimer's disease, atherosclerosis, heart failure, myocardial infarction, schizophrenia, and chronic fatigue syndrome (Figure 2) [56–58].

In addition, ROS has a big role in balancing the intracellular and extracellular Ca^2+^ levels [54, 59]. Cellular Ca^2+^ content is also known as one of the most versatile signals for the activation of protein kinase C (PKC) signal transduction cascades which are involved in the control of cellular processes and functions, such as contraction, secretion, metabolism, gene expression, cell survival, and cell death as well as the maintenance of plasma membrane fluidity by proton motive force [60] (Figure 2). ROS with free radical groups such as superoxide anion (O_2_^·−^), hydroxyl radical (^·^OH), and peroxynitrite (ONOO^−^) and the cellular Ca^2+^ level are well known to maintain the redox homeostasis and signaling events during normal physiological processes. Therefore, it is considered that the interaction between ROS and Ca^2+^ can be bidirectional [59–62]. Excessive and uncontrolled ROS signal or oxidative stress directly damages the plasma membrane fluidity and redox homeostasis [61, 62]. As a result, Ca^2+^ influx into the cytoplasm from the extracellular environment and disrupts the ion exchanging balance between the intra- and extra-cellular plasma membranes as well as mitochondria, which then leads to cell death or the apoptotic pathway by increasing the cytochrome c protein, which then in turn activates caspase 3 and caspase 9. The accelerated Ca^2+^ level disrupted the Ca^2+^ signal in the cellular cytoplasm, which dephosphorylates the protein and modulates the PKC signal transduction cascades, and is associated with many diseases and the aging process [60–62].

**Figure 3:**
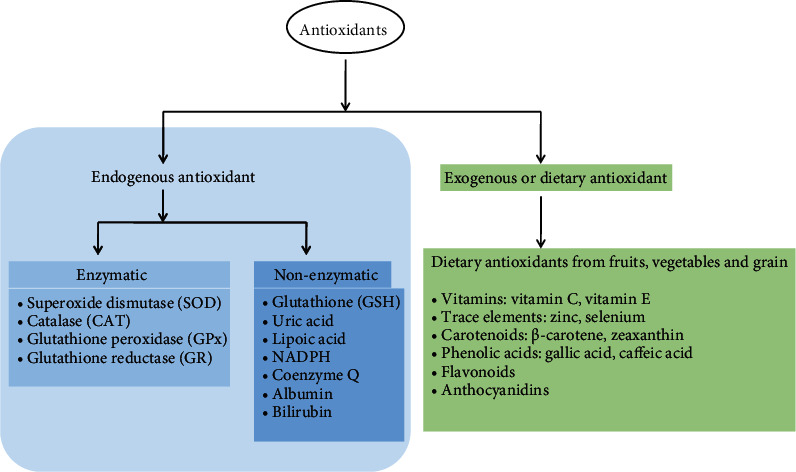
Classification of enzymatic and nonenzymatic antioxidants.

Primarily, antioxidants have the greatest defensive role in protecting the cell against oxidative damage [63]. Generally, antioxidants have a vital role in keeping optimal cellular functions and systemic health and well-being. However, under oxidative stress conditions, endogenous antioxidants in humans, although highly efficient, are not sufficient to protect the cell from the harmful effects of ROS [64]. Therefore, dietary antioxidants are required to maintain the optimal cellular defensive functions. The most efficient enzymatic antioxidants contain glutathione peroxidase (GPx), catalase (CAT), and superoxide dismutase (SOD), present in cellular cytoplasm and mitochondria. Nonenzymatic exogenous antioxidants are mainly derived in nature from photosynthetic organisms, fruits, vegetables, and plants and belong to different families such as vitamins E and C, thiol antioxidants (glutathione, thioredoxin, and lipoic acid), melatonin, carotenoids, and natural flavonoids [64–67] (Figure 3).

**Figure 4:**
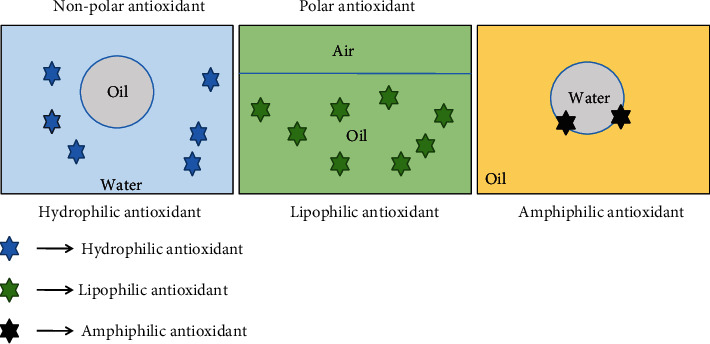
Efficacy of active antioxidants at different phases.

Antioxidants are considered as stable molecules that can donate an electron to a free radical and neutralize them, thereby scavenging the free radical and stopping the free radical from causing future damage [68]. Originally, antioxidants protect the cell from oxidative stress by applying one of the two mechanisms such as (a) the chain-breaking mechanism by which the primary antioxidant donates an electron to the free radical. (b) the second mechanism is the removal of ROS/reactive nitrogen species (RNS) initiators (secondary antioxidants) by quenching a chain-initiating catalyst. Antioxidants may provide their defensive action on biological systems at different levels including electron donation, metal ion chelation, radical scavenging, and repair or by gene expression regulation as well as prevent lipid peroxidation [69]. Moreover, antioxidants protect the cells by applying their strong multiphase efficacy system into the cell and cell membrane. It can strongly diffuse in both the aqueous and oil phases because of its polar and nonpolar paradox systems [70], known as a lipophilic, hydrophobic, and amphiphilic antioxidant (Figure 4). The efficacy of lipophilic antioxidants or polar antioxidants is to be more efficient than nonpolar or hydrophilic antioxidants. The lipophilic antioxidant can orient itself to the oil-water interface, where lipid peroxidation is induced whereas the hydrophilic antioxidant diffuses in the water phase, and therefore, it is less efficient [71]. Additionally, their activity also depends on the concentration of antioxidants, free radical types, and both the chemical structure and the reaction condition [69].

Nowadays, a large number of natural antioxidant compounds such as carotenoids, ascorbic acid, flavonoids, and phenolic compounds are derived from seaweed sources [22, 72–75]. The antioxidative efficiency of seaweeds is higher than that of plants and fruits [76–78]. It is already well reported that the brown seaweeds are the richest sources of these antioxidant compounds with a unique structure, which has a greater level of hydrophilic and lipophilic nature [14, 17, 79] (Table 2).

**Figure 5:**
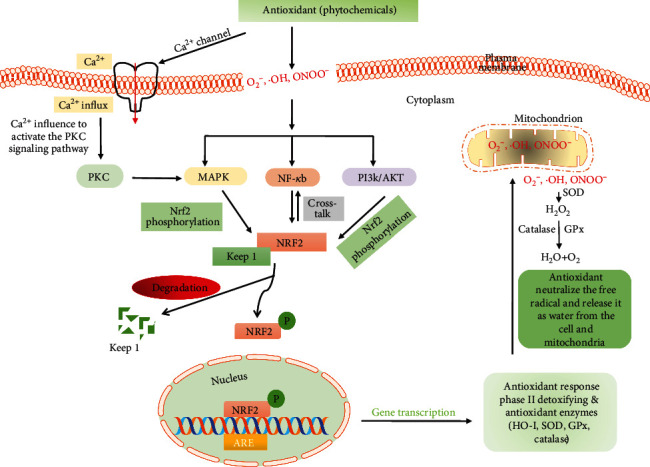
Antioxidant mechanism in the regulation of the Nrf2 signaling pathway with the crosstalk of the different signaling pathways.

The redox equilibrium plays a pivotal role in the cell's physiological and pathological functions by balancing the ROS and antioxidant as well as ROS stability. ROS stability needs to activate or deactivate a variety of receptors, proteins, ions, and other signaling molecules [50, 53]. The excessive accumulation or depletion of ROS leads to instability of the redox balance that may influence many cellular signaling pathways and confers the cellular dysfunction as well as subsequently developing various pathologies [50]. To equilibrate the redox balance, antioxidants not only produce the antioxidant but also have a great ability to modulate the ROS sensitive cell signaling with the activation of the antioxidant responsive element ARE/Nrf2 pathway [54, 55]. However, numerous studies have already elucidated that the phytochemicals or natural compounds of antioxidants successively modulate the ROS-sensitive signaling pathway and restock the antioxidant into the cell through maintaining the redox balance [86]. The activation of such signaling pathways increased the expression of gene-encoding cytoprotective proteins, including phase II enzymes, antioxidant enzymes, growth factors, and proteins involved in the regulation of cellular energy metabolism. The activation of the Nrf2 pathway intersects with other intracellular signaling pathways such as the MAPK, PI3k/Akt, and NF-*κ*B pathways [54, 55, 87] (Figure 5)

On the other hand, antioxidants actively reduce the cellular ROS which may help to balance the cellular redox balance as well as the intra- and extracellular cytoplasmic Ca^2+^levels [87]. Antioxidants reduce the intracellular ROS level by balancing the ionic exchange between the plasma membrane and the cytoplasmic membrane as well as modulate the Ca^2+^ channel called IP inositol 1,4,5-trisphosphate receptor (IP3R) [54, 55, 59, 87]. As a result, the Ca^2+^ ion binds to the PKC enzyme and activates the PKC signaling cascades, as well as activates various signaling pathways and also triggers the release and translocation of Nrf2 from the cytosol to the nucleus, which is responsible for the expression of antioxidant genes, thus maintaining the cellular redox homeostasis [55, 59] (Figure 5).

Brown seaweeds are the largest type of macroalgae that belong to the phylum of Phaeophyta, which means “dusky plants.” Naturally, it is brown or yellow-brown and found in temperate or arctic waters. Brown algae typically have a root-like structure called a “holdfast” to anchor the algae to a surface. There are about 1500-2000 species of brown algae worldwide [3]. The huge biodiversity of algae and the richness of physiological traits with their unique adaptive properties are considered as a potential target in the research area [88]. Most of the algae are oxygenic autotrophs; they can quickly and continuously adapt themselves to extreme environments by synthesizing their bioactive compounds with protective functions, limiting or repairing potential damage from a harmful environment. These bioactive compounds act as primary and secondary sources of metabolites that provide the antioxidant and other bioactive functions as well as maintain their cellular homeostasis [3, 88]. It has been already proven in numerous research and clinical studies that the compound of brown algae species has antioxidative activity [23–25, 78, 81].

**Figure 6:**
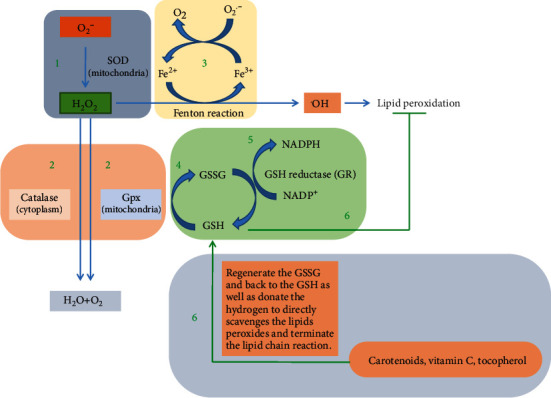
The enzymatic and nonenzymatic antioxidant protection mechanism. (1) Superoxide radical (O_2_^·−^) is formed by a single-electron reduction of oxygen. In a reaction catalyzed by superoxide dismutase (Cu/ZnSOD or MnSOD), superoxide radical binds an electron, which leads to the formation of hydrogen peroxide (H_2_O_2_). (2) In the further reduction of hydrogen peroxide (H_2_O_2_) to water and oxygen by catalase (CAT) and glutathione peroxidase (GPx) enzymes. (3) In the Fenton reaction, the H_2_O_2_ is then transformed into hydroxyl radical (HO^·^) by catalyzing the transition metal, which is further participating in the free radical chain reactions. (4) H_2_O_2_ is reduced by glutathione (GSH) and produced glutathione disulfide (GSSH). (5) The glutathione disulfide is then reduced by glutathione reductase (GR) using the hydrogen of NADPH which is oxidized to NADP^+^. (6) Besides, the nonenzymatic antioxidants such as carotenoids, vitamin C, and tocopherol support the regeneration of GSSG and back to GSH. Vitamin C, carotenoids, and tocopherol donate the hydrogen to free radicals that directly scavenges the free radical and terminates the lipid peroxidation chain reaction [66, 91, 92].

A large number of enzymatic and nonenzymatic antioxidants have been involved to detoxify the ROS and prevent the formation of highly reactive radicals such as hydroxyl radical (^·^OH) [88]. Enzymatic antioxidants mainly produced in a cell are usually high molecular weight substances such as SOD, glutathione reductase (GR), CAT, and ascorbate peroxidase. Primarily, enzymatic antioxidants inactivate the ROS intermediates and detoxify the ROS by supplying the NADPH for proper functioning (Figure 6). On the other hand, the dietary substances known as exogenous antioxidants (such as carotenoids, flavonoids, phenolic compounds, and ascorbic acid) scavenge the free radicals to break down the chain reaction responsible for lipid peroxidation [66–69]. The compounds of brown algae such as carotenoids, polyphenols, sulfated polysaccharides, and fucosterols have belonged as exogenous antioxidants, which may apply two defensive actions to cope with ROS before they can damage the cellular deference mechanism [89, 90] such as

1. The primary action is to break down the chain reaction, which results in free radicals becoming less reactive [66, 69]
2. The second mechanism is scavenging activity against superoxide and hydroxyl radicals by chelating/deactivating metals. The metal-binding proteins (such as albumin, ferritin, and myoglobin) inactivate the transition metal ions (such as copper, iron, manganese, zinc, and selenium act to upregulate the antioxidant enzyme activities) that catalyze the production of free radicals, quenching/scavenging the singlet and triplet oxygen (highly toxic) and removing ROS [66, 91, 92].

## 3. Antioxidant Effects of Brown Seaweed-Derived Compounds

### 3.1. Carotenoids

**Figure 7:**
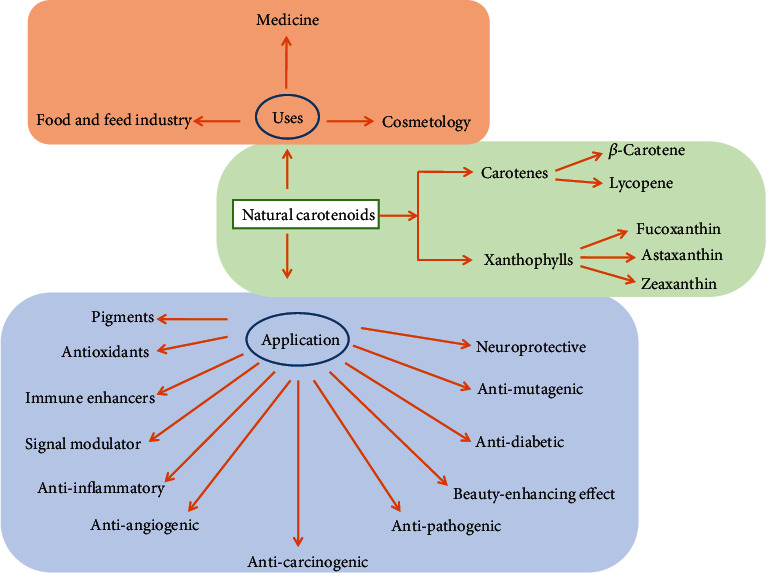
Distribution, biological functions, and application of natural carotenoids derived from brown seaweeds.

Carotenoids are a family of pigmented compounds that are naturally synthesized by plants, algae, fungi, bacteria, and archaea [29]. Over 1100 naturally occurring carotenoids are produced from those sources but not from animals [93]. Carotenoids are hydrophobic highly conjugated 40-carbon (with up to 15 conjugated double bonds) molecules. About more than 500 species of natural carotenoids are known and categorized into two groups: primary carotenoids which have pure hydrocarbons (those without any oxygen molecule), e.g., *α*-carotene, *β*-carotene, and lutein, and secondary carotenoids which are carbon (C), hydrogen (H), oxygen-containing xanthophylls (e.g., fucoxanthin, astaxanthin, canthaxanthin, and echinenone) are generally produced during the photosynthesis of seaweeds after exposure to specific environmental stimuli such as light and osmotic shock [39, 94–96]. The brown seaweeds are the rich source of carotenoids, which have been reported as powerful antioxidants with numerous beneficial health effects (Figure 7) [96, 97]

Carotenoids (Crt) are very potent natural antioxidants. The antioxidant actions of carotenoids are based on their singlet oxygen quenching properties and the ability to trap free radicals, which mainly depend on the number of conjugated double bonds [98]. Furthermore, carotenoids can scavenge oxidizing free radicals via three primary reactions (Equations (1) to (3)), by its addition, electron transfer, addition, and hydrogen atom transfer.

The carotenoid antioxidant reactions are as follows: (i) electron transfer between the free radical (R^·^) and Crt, resulting in the formation of a Crt radical cation (Crt^·^+) (Equation (1)) or Crt radical anion (Crt^·^−) (Equation (2)); (ii) they can transfer the electrons forming a radical cation (RCrt^·^) (Equation (3)); and (iii) hydrogen atom transfer leading to a neutral Crt radical (Crt^·^) (Equation (4)) [38, 99].

(1) $$\begin{array}{c} {\text{R}}^{\cdot }+\text{Crt}⟶\text{R}-+{\text{Crt}}^{\cdot }+\left(1\right)\left[\text{Addition}\right] \end{array}$$

(2) $$\begin{array}{c} {\text{R}}^{\cdot }+\text{Crt}⟶\text{R}++{\text{Crt}}^{\cdot }-\left(2\right)\left[\text{Addition}\right] \end{array}$$

(3) $$\begin{array}{c} {\text{R}}^{\cdot }+\text{Crt}⟶{\text{RCrt}}^{\cdot }\left(3\right)\left[\text{Electron transfer}\right] \end{array}$$

(4) $$\begin{array}{c} {\text{R}}^{\cdot }+\text{Crt}⟶\text{RH}+{\text{Crt}}^{\cdot }\left(4\right)\left[\text{Hydrogen ion transfer}\right] \end{array}$$

Generally, the principal defense process in carotenoids is their lipophilic nature that allows them to penetrate through the cellular lipid bilayer membrane and cross to the blood-brain barrier, carrying out its biological function also in different regions of the human body, including the brain [39]. Because as a lipid-soluble molecule, carotenoids actively scavenge the lipid and aqueous phase radicals, mostly they are located in the apolar core of lipid membranes. Thus, carotenoids are associated with various types of membranes within the cell such as the outer cell membrane, but also the mitochondria and the nucleus. They also work in lysosomes. As a consequence, carotenoids play a major protecting role in the cell membranes and lipoproteins from damage by free radicals [100].

**Figure 8:**
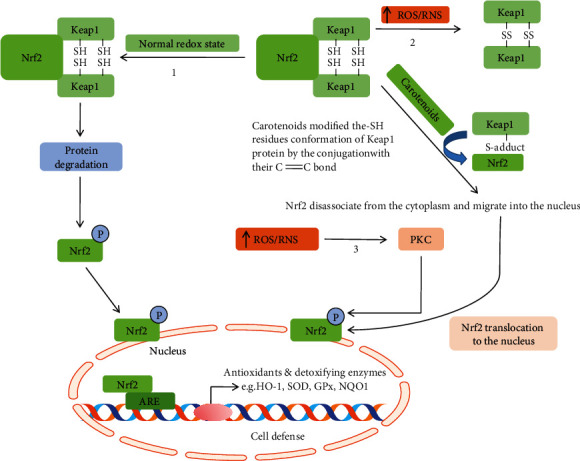
Electrophile activation of carotenoids of the Keap1-Nrf2 systems.

Several evidences suggested that carotenoids can act as redox agents in the case of oxidative stress due to their electrophile character and their conjugated double bonds that enhance the endogenous antioxidant Nrf2-Keap1 systems [101] which eventually help to maintain the redox homeostasis [80]. The redox homeostasis mainly depends on the nucleophile compounds, e.g., glutathione (GSH) and thiol (–SH) as well as their reductase enzymes GR, heme oxygenase-1 (HO-1), glutathione S-transferases (GSTs), and NAD(P)H quinine oxidoreductase 1 (NQO1). The GSH or –SH (thiol-) containing enzymes play a pivotal role in the free radical chain reaction that promotes to sustain the redox homeostasis (Figure 6) [102, 103]. Studies demonstrated that the carotenoids could increase the GSH level through the modulation of redox-sensitive –SH groups of Kelch-like ECH-associated protein 1 (Keap1). The Keap-Nrf2 signaling pathway plays an important role in the cellular defense against oxidative stress [104, 105]. Generally, under the normal physiological condition, Nrf2 is bound with their repressor protein Keap1 in the cytosol; which leads to its degradation by the ubiquitinylation process. Keap1 is a cysteine-rich protein, and its –SH residue conformation can be modified by different oxidants and electrophiles, thereby leading to the liberation of Nrf2 and transfer to the nucleus [80, 106]. At a higher oxidative stress condition, excessive ROS promotes the disassociation of Keap1 and Nrf2 either via the activation of PKC that assists to phosphorylate the Nrf2 or by oxidation of the cysteine residues of Keap1 that mainly govern the Keap1 activity [54–57, 107]. The carotenoids act as antioxidants through their electrophile C=C groups which conjugate with the aldehyde group (–CHO) of Keap1 modifying their thiol residues allowing the Nrf2 release into cytosol and translocation to the nucleus. Thus, Keap1 is inactivated, and newly synthesized Nrf2 binds to the ARE, which then leads to the expression of phase II enzymes and cytoprotective enzymes, e.g., GR, heme oxygenase-1 (HO-1), GSTs, NAD(P)H quinine oxidoreductase 1 (NQO1), SOD, and GPx [108] (Figure 8).

The scavenging function of carotenoids also has a signal-modulating role at the cellular signaling cascades such as the NF-*κ*B, MAPK [109, 110], and PI3/Akt survival pathways as well the caspase pathway [111, 112]. Several studies suggest that carotenoids can act as modulators of redox-sensitive signaling cascades also able to progress the cell cycle through the direct modulation of cell cycle-related proteins as well as different signaling pathways which are usually involved in cell proliferation [108]. According to the studies, it can be hypothesized that *β*-carotene can modify the cellular redox status by changing the intracellular antioxidant status [112]. Studies showed that it can also delay the cell cycle G2/M phase by decreasing the expression of cyclin A in human colon adenocarcinoma cells [113] with increase of the antioxidant level which might help in decreasing the apoptotic protein as well as modifying the cellular growth [111]. It has been also reported that cell cycle progression and differentiation are dependent on the carotene dose, because it has a prooxidant role under high oxygen tension [113, 114]. Several indications suggested that carotenoids may also alter the expression of the apoptosis-related protein including the Bcl-2 and the caspase protein by a redox mechanism [111, 115]. Free radical species such as singlet oxygen [116] and nitric acid [117] have been reported to activate the caspase 8, an important protein degrading enzyme involved in the apoptotic cascade [108], whereas studies found carotenoids can initiate the caspase 3 activities in several cell lines, mainly by interacting with a single complex located on the cell membrane and inducing caspase 8. It was also reported that carotenoids protect the colon cancer cell from apoptosis by decreasing the expression of antiapoptotic protein Bcl-2 [112]. Such effects are mainly strictly related to apoptosis induction and ROS production by the carotenoids. This finding interestingly demonstrated that carotenoids have a role in an antioxidant pathway, whereby this protein can prevent programmed cell death by decreasing the ROS formation and lipid peroxidation products [115]. *In vitro* studies observed that *β*-carotene may increase the expression of the proapoptotic protein Bax in U-937 cells and HUVEC cells [112].

**Figure 9:**
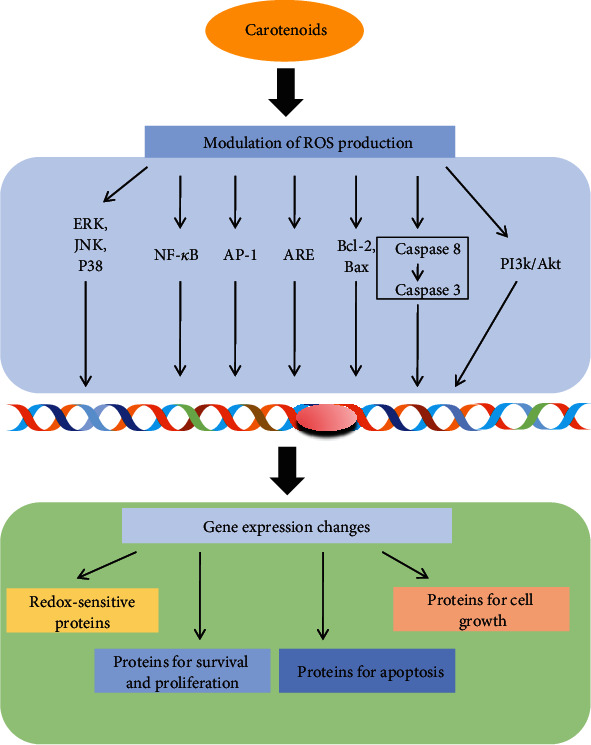
Scheme representing the multitude effects of carotenoids on the ROS-dependent signaling pathway and their related target protein gene expression. Carotenoid molecules may modulate the intracellular ROS production and can activate the redox-sensitive transcription factors or directly affect DNA damage which in turn may modify the gene expression.

The NF-*κ*B pathway is generally thought to be a primary oxidative-response pathway [112]. Studies have shown that carotenoids can inhibit the oxidative stress-induced NF-*κ*B pathway with the addition of their electrophile groups and the cysteine residues of I*κ*B kinase IKK and NF-*κ*B subunits (p65) [118]. It has been reported that *β*-carotene prevents the phosphorylation of ERK, JNK, and P38 in oxidative stress [119]. Recently, it has been reported that *β*-carotene may affect cellular growth through the modulation of redox-sensitive transcription factor AP-1 [112]. Carotenoids may also modulate the antioxidant response regulatory gene (ARE) and can alter the gene expressions of phase II enzymes (i.e., HO-1, GSTs, NQO1, SOD, and GPx), resulting in cellular redox homeostasis [112] (Figure 9).

Some of the studies indicate that carotenoids can also modulate oxidative stress-induced calcium signaling [120]. Carotenoids can decrease the calcium/calmodulin-dependent protein kinase IV (CAMKIV) enzyme by binding with the active site of CAMKIV; that form allows the cancer cell apoptosis [121]. CAMKIV is an enzyme belonging to the Ser/Thr kinase family. Generally, it plays a role in cell proliferation, migration, angiogenesis, and inhibition of apoptosis as well as calcium-dependent cell signaling. Elevated intracellular calcium ion concentration makes a complex form between Ca^2+/^calmodulin, which induces the phosphorylation of the transcription factor and causes various types of cancer [122, 123].

**Figure 10:**
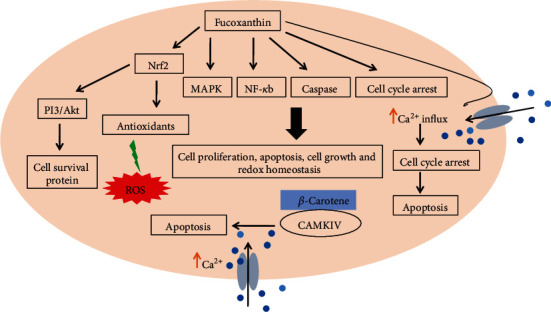
Carotenoids and fucoxanthin effects on the cellular calcium level along with the multitude effects of fucoxanthin on cellular signaling pathways. Fucoxanthin protects the cell from oxidative stress by modulating several cell signaling pathways.

One of the well-known carotenoids is fucoxanthin, considered as a potential antioxidant because of its unique chemical structure including an allelic bond (C=C), epoxide group, and hydroxyl group [124]. It is well-known, and unique structural carotenoid pigments are present in brown seaweed that have not been found in other carotenoids [35, 125]. Fucoxanthin accounts for around 10% of the total natural production of seaweed carotenoids [126], which shows strong antioxidative properties by the inhibition of intracellular ROS formation, DNA damage, and apoptosis as well as exhibiting strong enhanced cell viability against H_2_O_2_-induced oxidative stress [127]. Several studies have demonstrated their experiments that fucoxanthin is safe and nontoxic to cells even at repeated doses [128–131]. *In vivo* studies also have shown that a fucoxanthin diet elevated the antioxidant activities including CAT, SOD with the mRNA expression of Nrf2 and its target genes such as NQO1 [132]. It has an active role in the modulation of signaling pathways including MAPK [133], NF-*κ*B [134], and apoptosis caspase pathways [135] as well as induces cell cycle arrest [136]. Recently, it was shown that fucoxanthin suppresses H_2_O_2_-induced inflammation and oxidative damage in microglial cells via the attenuation of the phosphorylation of the MAPK signaling pathway, as well as by the free radical scavenging capacity of fucoxanthin and its ability to regulate the endogenous antioxidant system [137] It can also exert the cytoprotective effects against H_2_O_2_-induced oxidative stress through the activation of the PI3K-dependent Nrf2-signaling pathway along with the expression of mRNA and protein relative cytoprotective gene LO2 cells [138]. Sangeetha and collaborators *in vivo* analyzed the properties of fucoxanthin compared with *β*-carotene [139]. These two carotenoids decreased lipid peroxidation as well as enhanced the CAT and GST activities, showing the protective effect against Na^+^K^+^-ATPase activity. However, fucoxanthin exhibited higher antioxidant and protection properties than *β*-carotene [39, 139]. Many studies suggested that fucoxanthin increases the intracellular cytosolic Ca^2+^ that triggers cell apoptosis through modulation of the cell cycle arrest [135, 136] (Figure 10).

### 3.2. Polyphenols and Phlorotannins

Algal polyphenols are a large and diverse class of secondary metabolites that consist of around 8000 naturally occurring compounds that possess vital biological functions including antioxidant and free radical scavenging properties [27, 140]. These biological properties mainly shared by this molecule are their phenol groups [27]. The antioxidant radical scavenging activity of these compounds generally precedes through the hydrogen atom transfer or electron transfer mechanisms [141–143]. The polyphenol compounds have one or more hydroxyl groups (-OH), which directly bond to a phenolic or aromatic hydrocarbon group, and the hydrogen atom transfer mechanism indicates the ability of the phenolic derivatives (ArOH) to transfer an -H atom from the phenolic -OH group [144]. Generally, the stability of the resulting phenolic radicals (ArOH^·^) governs the radical scavenging ability of these compounds [27].

(5) $$\begin{array}{c} {\text{R}}^{\cdot }+\text{ArOH}-=\text{RH}+\text{ArO}\cdot \end{array}$$

In the electron transfer mechanisms, the ability to transfer an electron governs the radical scavenging activity of phenolic derivatives. The stability of these radical cations depends on their ionization potential. The lower potential value indicates the better stability of the electron [27].

(6) $$\begin{array}{c} {\text{R}}^{\cdot }+\text{ArOH}-={\text{ArOH}}^{\cdot +}+R^{-} \end{array}$$

**Figure 11:**
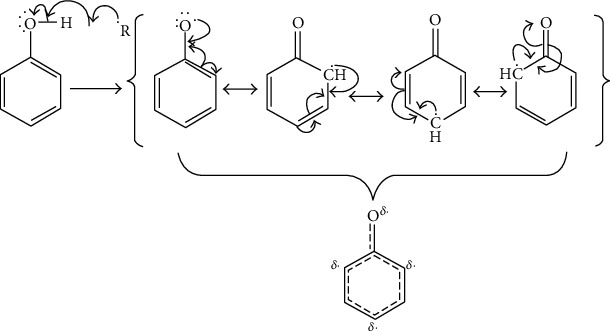
The proposed reaction between phenol and a free radical, which undergoes resonance stabilization; indicates the radical scavenging ability of compounds (i.e., curved half-headed arrows represent the transfer of a single electron).

Given the simplified reaction mechanism (Figure 11), phenols can react with free radical species (R^·^) giving phenolic radicals that undergo resonance stabilization within the molecule by delocalizing the unpaired electron within the aromatic ring leading to a stable intermediate (indicated by the resonance hybrid). This resonance stabilization stabilizes the radical scavenging ability of phenol compounds [27]

**Figure 12:**
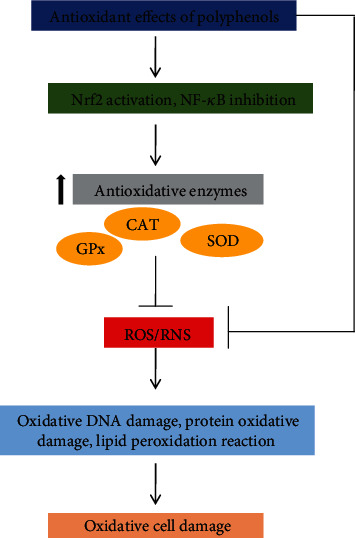
Potential mechanisms of polyphenols to the protection of the cell against oxidative stress.

**Figure 13:**
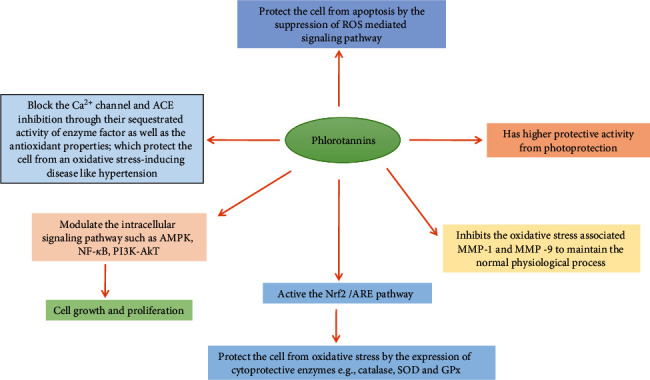
The biological role of phlorotannins on the function of the cell signaling pathway.

The antioxidant effect of polyphenols has also the ability to enhance the enzymatic activity of different enzymes, including catalase, glutathione peroxidase, and superoxide dismutase, by their potent free radical scavenging properties and their ability to interact with other molecular targets, as they are capable of activating the Nrf2/ARE pathway [145] (Figure 12). Among algal polyphenolic compounds, phlorotannins have also been shown to have a high antioxidant property because of their highly hydrophilic components and the presence of –OH groups that can form hydrogen bonds with water. They have a wide range of molecular sizes between 126 and 650 kDa and can occur in various concentrations (0.5-20%) in brown algae [27, 146]. Brown microalgae accumulate a variety of phloroglucinol-based polyphenols as phlorotannins of low, intermediate, and high molecular weight containing both phenyl and phenoxy units, whichever can also be linked with each other or other algae such as red algae. Based on this linkage, phlorotannins can be classified into 4 major subclasses: (a) fuchalos and phlorethols (phlorotannins with an ether linkage), (b) fucols (with a phenyl linkage, (c) fucopholoroethols (with an ether and phenyl linkage), and (d) eckols (with an ether and phenyl linkage) [146]. Pholorotannins isolated from *Ecklonia cava* and their compounds (phlorofucofuroeckol A, dieckol, phloroglucinol, eckol, 7-pholoroeckol, 2-phloroeckol, and fucodipholoroethol G) are one of the most abundant sources of polyphenolic that have been mostly reported of biological activities including anti-inflammatory, antidiabetic, anticancer, antimicrobial, and antiviral, whereas most of them are focused on antioxidant activity [20, 146, 147]. Many researchers have shown that the eckol, phlorofucofuroeckol A, dieckol, and 8,8′-bieckol have shown potent inhibition of phospholipid peroxidation at 1 *μ*M in a liposome system [147, 148], and these phlorotannins have significant radical scavenging activities against superoxide and DPPH radicals effectively compared with ascorbic acid and *α*-tocopherol [09]. There are several pieces of research that have shown that *Ecklonia cava*-derived phlorotannins and their bioactive compounds can modulate the ROS-mediated intracellular signaling pathway as well as gene expression and inhibit the enzymes [20, 145, 149, 150], which eventually protect the cellular damage from oxidative stress and apoptosis [20]. Studies also showed the phlorotannins may also protect the cancer cell by the inhibition of ROS-generating MMP-2 and MMP-9 via the NF-*κ*B pathway [151]. It has been reviewed that phlorotannins can block the Ca^2+^ channel as well as reduce the Ca^2 +^ influx which are stimulated by ROS (Figure 13) [20]. Due to several profound capabilities of *E. cava*, derived compounds are interesting to use as functional ingredients in pharmaceuticals and cosmeceuticals [147, 152, 153]

### 3.3. Sulfated Polysaccharides

**Figure 14:**
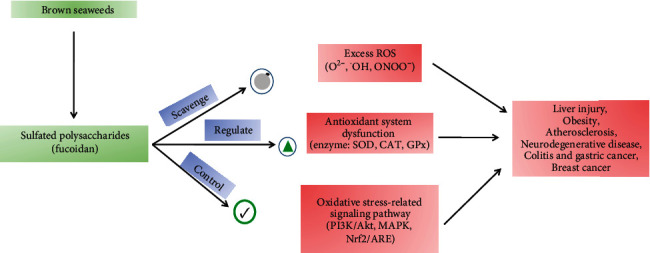
Overview of sulfated polysaccharide (fucoidan) derived from brown seaweeds in alleviating oxidative stress-mediated disease.

Marine SPs are polymeric carbohydrates with large hydrophilic molecules. They have a fundamental role in living marine organisms such as energy storage and protection or as a structural molecule, which are mainly produced by photosynthesis [85]. The algal polysaccharides possess potent bioactivities because of their unique physicochemical properties, such as high content of fucose, galactose, uronic acid, and sulfate [154]. Microalgae can accumulate carbohydrates to more than 50% of their dry weight, due to the high photoconversion efficiency of the photosynthetic process [155]. These compounds are present in high concentrations and vast diversity in microalgae [156]. A vast number of researchers have reported that the SPs and its compounds could alleviate oxidative stress-mediated diseases, such as liver injury, diabetes, obesity, neurodegenerative disease colitis, and cancer [155–161]. This effect could be explained by three distinct mechanisms, including scavenging the ROS and regulating the antioxidant system or oxidative stress-mediated signaling pathways and also implying the complicated interactions of marine-derived antioxidant polysaccharides in reducing the oxidative stress [46, 47] (Figure 14). The antioxidant activity of SPs depends on their structural property, such as the degree of sulfating, their molecular weight, types of the major sugars, and glycosidic branching [46, 162]. The low molecular weight SPs have been shown to be potent antioxidants rather than the high molecular weight SPs [163]. It has been also reported that low molecular weight (~30 kDa) SPs can promote cell proliferation [164] as well as cell protection from the oxidative stress-induced apoptosis, which are directly and indirectly related to their antioxidant properties [46, 165]. Currently, SP has become a hot research area in the field of regenerative medicine and tissue engineering application due to its unique structure and specific features as antioxidants, antitumors, immunomodulatory, inflammation, anticoagulant, antiviral, antiprotozoal, and antibacterial [46, 47, 162, 165].

Among the three types of SPs extracted from seaweeds, the brown seaweeds are the richest source of antioxidants [46, 47]. The major bioactive SP extracted from brown seaweeds is fucan which is usually defined as fucoidan; it is water-soluble and composed of significant quantities of L-fructose and sulfated ester groups accompanied with a little percentage of other monosaccharides, e.g., arabinose, xylose, glucose, galactose, and mannose. These constitutes of polysaccharides are known to be one of the major components of the brown algae cell wall that protects the cell in stress or low tide environments [46, 165].

**Figure 15:**
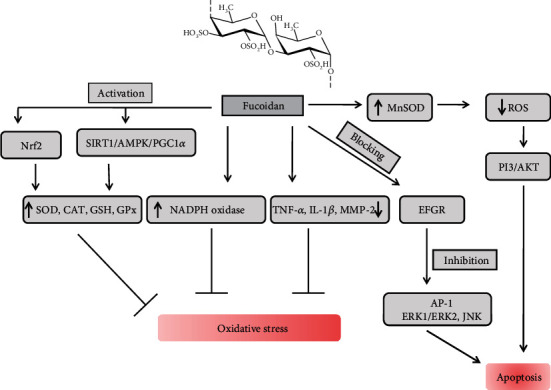
Fucoidan prevents oxidative stress and cell apoptosis by regulating the antioxidant system and signal-modulating pathway.

Numerous *in vivo* and *in vitro* studies have revealed that fucoidan derived from brown algae can alleviate body damage from oxidative stress through the regulation of the antioxidant system in the body [45, 165, 166]. The excellent antioxidant activity of fucoidan is mainly dependent on their desulfation or oversulfation that allows the development of derivatives of fucoidans [46, 165, 166]. The DPPH free radical scavenging assay is widely used to evaluate the *in vitro* antioxidant activity [167]. Polysaccharides provide the hydrogen or electrons to DPPH free radicals to form stable molecules (DPPH-H) [168]. The electron-withdrawing group of polysaccharides and the specific structures activate the hydrogen atoms on sugar residues [169]. Many studies have already found that sulfated polysaccharides from brown algae have strong DPPH free radical scavenging activity and reduction ability. Furthermore, SPs also have strong ferrous ion-chelating and reducing power activities [170, 171]. Many studies have revealed that fucoidan inhibits oxidative stress by downregulating the malondialdehyde (MDA) level and upregulating the SOD level [172]. Moreover, studies have documented that fucoidan extract can reduce the levels of lipid peroxidation markers MDA and TBARS in alcoholic rats and increase the levels of the nonenzymatic antioxidant GSH and the enzymatic antioxidant SOD, CAT, and GPx in the liver, ultimately reducing the oxidative damage caused by alcohol. In addition, fucoidan also reduces the mRNA expression of TNF-*α*, IL-1*β*, and MMP-2 to inhibit the production of ROS in the liver, thus alleviating the development of nonalcoholic fatty liver disease [173]. Similarly, fucoidans inhibit the proliferation and induced apoptosis through the downregulation of the PI3K/AKT pathway as well as the reduction of Bcl-2 and Bcl-xl and the rise of the expression of Bax, caspase 3, and caspase 9 activation [174]. It can also inhibit the epidermal growth factor (EGF) signaling in several cancer cell types, by preventing binding their receptor site that promotes to block the activation of activator protein-1 (AP-1) which also diminishes the transactivation activity of ERK1/ERK2 and JNK signaling protein [175]. Another study also showed that fucoidan can inhibit apoptosis and improve the cognitive ability in Alzheimer's disease model mice by upregulating the expression of the apoptosis-inhibiting proteins by activating the SOD activity and increasing the GSH levels [176]. Recently, a study found that fucoidan can improve atherosclerotic cardiovascular disease by reducing the production of ROS by inhibiting NADPH oxidase (Figure 15) [177]. Besides, fucoidan can also inhibit ROS production through the regulation of the antioxidant defense system, thereby alleviating oxidative stress-related diseases. A study revealed that low molecular weight fucoidan regulates the activity of SOD and CAT by activating the SIRT1/AMPK/PGC1*α* signaling pathway, inhibiting the superoxide production and the lipid peroxidation, reducing TNF-*α* and transcription factor NF-*κ*B, and then inhibiting oxidative stress in the liver (Figure 15) [164]. Furthermore, fucoidan significantly increased the expression of MnSOD and decreased the ROS level through the PI3/AKT pathway and finally enhanced the survival and angiogenesis of mesenchymal stem cells in the ischemia model [178]. Interestingly, it was reported that it could upregulate the expression of HO-1 and SOD-1 genes by activating Nrf2 and then attenuate the oxidative stress in HaCaT cells [163]. Research also showed that the *Turbinaria ornata* protects the human carcinoma cell through the modulation of PPAR gamma, NF-*κ*B, and oxidative stress [179]. Another study also proved that the *Turbinaria ornata* protects the hepatic injury from azoxymethane-induced oxidative stress by exerting multiple pathways including the abolishment of inflammation and oxidative damage and the activation of PPAR gamma with increase of the antioxidant enzymes like SOD and GPx activities [180]. Thus, we could be review by all of these studies that fucoidan derived from brown algae not only has significant antioxidant activity but also modulates the oxidative stress-mediated diseases by regulating the antioxidant defense systems and the oxidative stress-related signaling pathway in cellular and experimental animal models [22].

### 3.4. Sterols

There are abundant sterols in brown seaweeds. These compounds occur in the free form, esterified with fatty acids, or are involved in glycosylated conjugates [49, 181]. Sterols are amphipathic and triterpene compounds that can reach about 5.1% of the total biomass in the microalgae [181, 182]. Most of them contain 28 or 29 carbons and 1 or 2 carbon-carbon double bonds, typically one in the sterol nucleus and sometimes a second in the alkyl side chain [181]. Moreover, fucosterol displays a high diversity of the unique compounds of phytosterols, such as brassicasterol, sitosterol, and stigmasterol [182, 183]. Some species contain a mixture of ten or even more phytosterols [183]. Sterol composition varies depending on the algae strain and can be modulated by light intensity, temperature, or growth phase [83]. Sterols have received much attention in the last few years because of their cholesterol-lowering properties [181]. Fucosterol is structural and functionally similar to cholesterol; however, it contains an alkyl; fucosterol, a phytosterol found in brown seaweeds, is well recognized for its various beneficial biological activities [49].

Studies showed that fucosterol elevates the activities of free radical scavenging enzymes such as SOD, CAT, and GPx against hydrogen peroxide. It can also restore the SOD and GPx cellular defensive enzymes which help to prevent cell membrane oxidation [48]. It can also suppress the nitric oxide (NO) and ROS generation through the suppression of inducible nitric oxide synthase (iNOS) and cyclooxygenase 2 (COX-2) expression because increasing iNOS and COX-2 is responsible for inflammatory disease [49]. Moreover, fucosterol exhibits inhibitory activity against acetyl- and butyryl-cholinesterases (AChE and BChE, respectively) [184, 185] and *β*-secretase enzyme which is responsible for Alzheimer's disease, although this enzyme production level is mainly associated with increasing the oxidative stress [186].

In the study of pharmacology and *in silico* analysis, it was revealed that the fucosterol action could intervene in the disease progression through modulating the biological processes as well as the signaling pathway, such as cytokine-mediated signaling pathway, apoptotic process, transcription regulation, inflammatory response, aging, response to lipopolysaccharide, NF-*κ*B activity, and also cellular response to ROS, which are closely associated with the disease pathophysiology. Studies showed that fucosterol could pass through the cell membrane to reach directly to various intracellular targets. The pharmacological network data reported that fucosterol showed a close association with the target proteins of many crucial pathways at the molecular and cellular levels [187]. Another report demonstrated that fucosterol attenuates the oxidative stress through the upregulation of antioxidant enzymes such as GPX1, SOD, CAT, and HO-1 via Nrf2/ARE and PI3/Akt signaling activation [49, 188].

**Figure 16:**
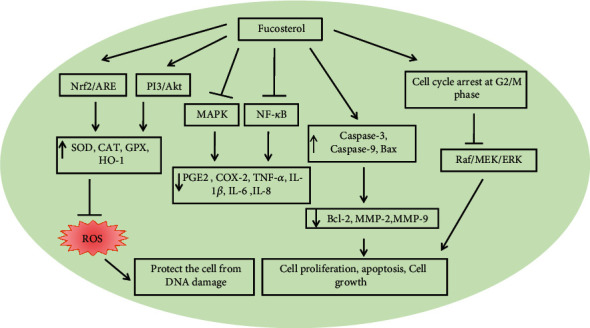
Overview of the biological role of fucosterol derived from brown seaweeds in alleviating the oxidative stress-mediated signaling pathway.

Fucosterol treatment can inhibit the oxidative stress-induced MAPK and NF-*κ*B signaling pathway along with reducing the levels of inflammatory mediators PGE2 and COX-2, as well as proinflammatory cytokines TNF-*α*, IL-1*β*, IL-6, and IL-8 [188]. Studies represented an excellent role of fucosterol in cancer cell proliferation, cell cycle, and apoptosis [189]. It was found that fucosterol causes the arrest of the cancer cells at the stage of the G2/M phase that prevents the cancer cells to enter the mitosis stage which can prevent cell growth and proliferation [190]. The Raf/MEK/ERK signaling pathway contributes to cancer cell proliferation. It also showed that fucosterol induced cell cycle arrest, via the inhibition of Raf/MEK/ERK signaling pathways that ultimately prevents cell proliferation [191]. Fucosterol can induce cancer cell apoptosis by increasing the expression of cleaved caspase 3, caspase 9, and Bax and decreasing the expression of Bcl-2, MMP-2, and MMP-9 [189, 192] (Figure 16). Research showed that the species of fucosterol named *Padina pavonica* exerted an excellent antidiabetic and antioxidant effect via the activation of PPAR*γ* mechanisms [193]. Fucosterol treatment can protect the cell from DNA damage after oxidative stress [188]

## 4. Conclusion

The research on brown seaweeds and their bioactive compounds' biological spectrum has been widely raised in recent years. Brown seaweeds and their derived compounds such as phlorotannins, SPs, fucosterol, and carotenoid pigments including fucoxanthin radical scavenging property are now clinically emphasized as a novel therapeutic compound. Antioxidants not only scavenge the free radical but also have numerous roles in the signal transduction pathway. From this point of view, we have attempted to gather plenty of information on how brown seaweeds and their antioxidant compounds modulate the oxidative stress signaling pathway as well as regulate the antioxidant system. However, the mechanisms of seaweeds are still obscure in the case of stress-causing disease. Thus, more studies should be focused on the investigation of activation and modulation as well as specific effects on the signaling pathway. The potentiality and structural features of the seaweed compound can have different effects on the cell. Additionally, long-term toxicity and drug delivery approaches should be evaluated.

## Figures and Tables

**Table 1:** Antioxidant activity of brown algal-derived compounds.

General category	Antioxidant compound	Antioxidant activity reported
Carotenoids	Fucoxanthin	[25, 35–39]
Polyphenols		
Phlorotannins	Phlorofucofuroeckol A, dieckol, phloroglucinol, eckol, 7 pholoroeckol, and 2-phloroeckol	[27, 40–43]
Sulfated polysaccharide	Fucoidans	[16, 32, 44–47]
Sterols	Fucosterol	[48, 49]

**Table 2:** Antioxidant efficacy of brown seaweeds compounds.

Antioxidant efficacy in the compound of brown seaweeds		
Lipophilic	Hydrophilic	Amphiphilic
Carotenoids [22, 78, 80]	Phlorotannins [22, 81, 82]	Sterols [22, 83, 84]
	Sulfated polysaccharide [22, 85]	
